# Supplementary material for: Hematopoietic stem and progenitor cell-restricted Cdx2 expression induces transformation to myelodysplasia and acute leukemia
Source: Nat Commun. 2020 Jun 15;11:3021. doi: 10.1038/s41467-020-16840-2 (PMC7296000; doi:10.1038/s41467-020-16840-2)
Supplement: Supplementary file 1 — Supplementary Information [file 41467_2020_16840_MOESM1_ESM.pdf]

**Supplementary Information**

# Hematopoietic stem and progenitor cell-restricted Cdx2 expression induces transformation to myelodysplasia and acute leukemia

Vu et. al.

**This file contains:**

Supplementary Figures 1-7

Supplementary Tables 1-5

Supplementary Methods

Supplementary References

# Supplementary Figure 1

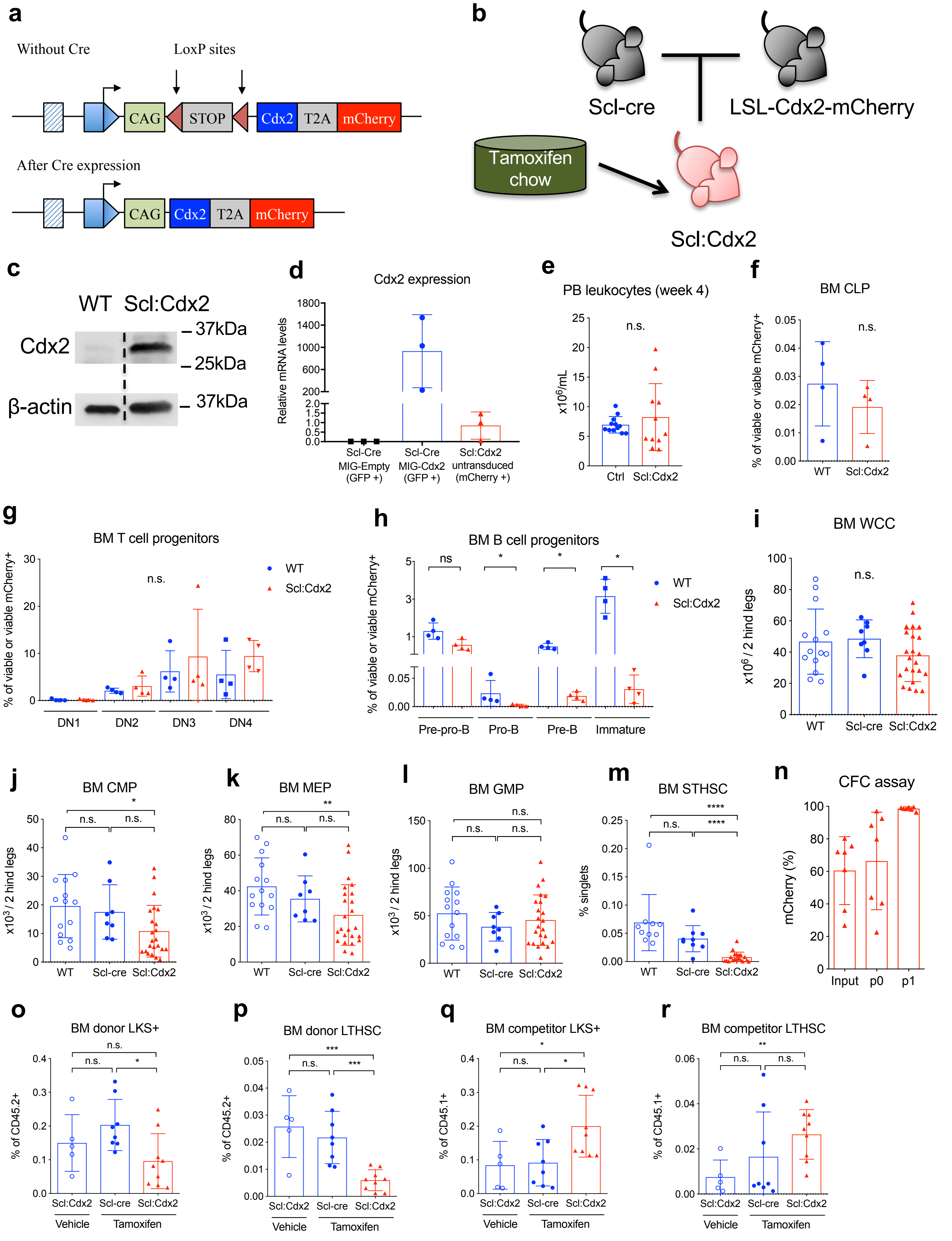

**Supplementary Figure 1. Cdx2 expression in HSPC alters progenitor subsets and self-renewal function.**

(a) Schematic of Cdx2-mCherry construct used to generate LSL-Cdx2-mCherry mice. Cdx2 and mCherry cDNA are separated by a T2A cleavage peptide and cloned downstream from a loxP-flanked stop codon. Cre expression excises the stop site activating expression of Cdx2 and mCherry proteins from a synthetic CAG promoter. (b) Diagram of breeding strategy to produce Scl:Cdx2 mice. LSL-Cdx2-mCherry mice are crossed with *Scl*-CreER$^T$ (referred to as *Scl*-Cre) and Scl:Cdx2 mice are fed tamoxifen-loaded chow for two weeks. (c) Immunoblot of Cdx2 and β-actin in *Scl*-Cre and Scl:Cdx2 bone marrow (BM) lysates four weeks after tamoxifen induction. Shown is representative blot from independent replicates, full image supplied in Supplementary Data 6. (d) qPCR of Cdx2 transcript levels relative to Actin and GAPDH. *Scl*-Cre cells were lineage-depleted and transduced with MSCV-IRES-GFP (MIG)-Cdx2 or MIG-Empty retrovirus and sorted for GFP. Scl:Cdx2 BM was induced with tamoxifen and sorted for mCherry. N=3 per group. (e) Leukocyte count of peripheral blood (PB) four weeks after tamoxifen induction (Ctrl n=12; Scl:Cdx2 n=11). Frequency of (f) common lymphoid progenitors (CLP), (g) T cell progenitors (gated on TCRβ$^+$; DN1, CD44$^+$CD25$^-$; DN2, CD44$^+$CD25$^+$; DN3, CD44$^-$CD25$^+$; DN4, CD44$^-$CD25$^-$), and (h) B cell progenitors (Pre-pro-B, B220$^+$CD19$^-$cKit$^-$IgM$^-$; Pro-B, B220$^+$CD19$^+$cKit$^+$IgM$^-$; Pre-B, B220$^+$CD19$^+$cKit$^-$IgM$^-$; immature B cells, B220$^+$CD19$^+$cKit$^-$IgM$^+$) in WT (n=4) or Scl:Cdx2 (n=4) BM. (i) BM white blood cell count (WCC) of WT (n=14), *Scl*-Cre (n-8) and Scl:Cdx2 (n=23) mice. Absolute cell counts of (j) common myeloid progenitors (CMP), (k) megakaryocyte-erythroid progenitors (MEP) and (l) granulocyte-macrophage progenitors (GMP) in BM (WT n=14; *Scl*-Cre n=8; Scl:Cdx2 n=23). (m) Frequency of short-term hematopoietic stem cells (STHSC) in BM (WT n=10; *Scl*-Cre n=9; Scl:Cdx2 n=19). (n) mCherry percentage of cells derived from colony forming cell (CFC) assay of Scl:Cdx2 BM cells (n=7). (o) Frequency of LKS+ and (p) LTHSC in CD45.2+ Scl:Cdx2 donor population, and (q) LKS+ and (r) LTHSC in CD45.1+ WT population in BM chimeras (Scl:Cdx2/Vehicle n=5; *Scl*-Cre/Tamoxifen n=8; Scl:Cdx2/Tamoxifen n=9). N = biologically independent animals. Statistical analyses performed using two-tailed Mann-Whitney test. Data are plotted as mean values +/- SD. n.s.; not significant. * $P < 0.05$, ** $P < 0.01$, *** $P < 0.001$, **** $P<0.0001$.

# Supplementary Figure 2

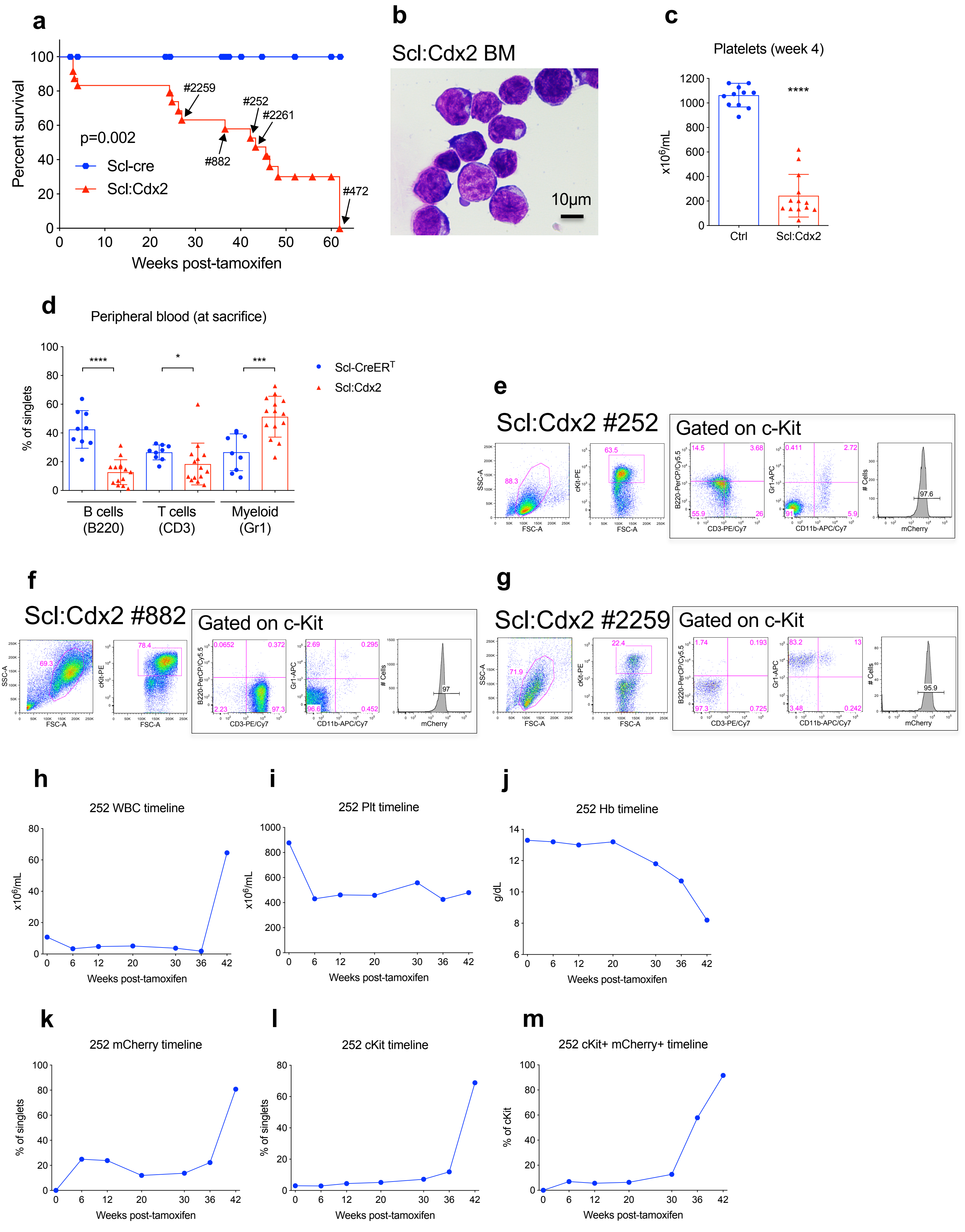

**Supplementary Figure 2. Cdx2 expression in HSPC results in lethal and transplantable disease.**

(a) Survival curve of tamoxifen-treated mice (*Scl*-CreER$^{T}$, n=18; Scl:Cdx2, n=22). Black arrows and identification numbers denote time points when acute leukemia mice were sacrificed. (b) Representative cytospin of leukemic blasts found in Scl:Cdx2 BM. Scale bar on bottom right of image indicates 10$\mu$m. (c) Platelet counts of mice four weeks after tamoxifen induction (n=12 per group). (d) Frequency of B220-positive B cells, CD3-positive T cells and Gr1-positive myeloid cells in PB of mice at sacrifice (*Scl*-cre n=9; Scl:Cdx2 n=14). Representative flow cytometry plots showing PB immunophenotype of leukemia-bearing mice (e) #252, (f) #882, (g) #2259. Time course of Scl:Cdx2 #252 PB (n=1) showing (h) WBC, (i) platelets, (j) hemoglobin, (k) mCherry, (l) cKit frequency and (m) cKit$^{+}$ mCherry$^{+}$ cell frequency. N = biologically independent animals. Statistical analyses performed using two-tailed Mann-Whitney test. Log-rank Mantel-Cox test used for survival curves. Data are plotted as mean values +/- SD. n.s.; not significant. * $P < 0.05$, *** $P < 0.001$. **** $P < 0.0001$.

# Supplementary Figure 3

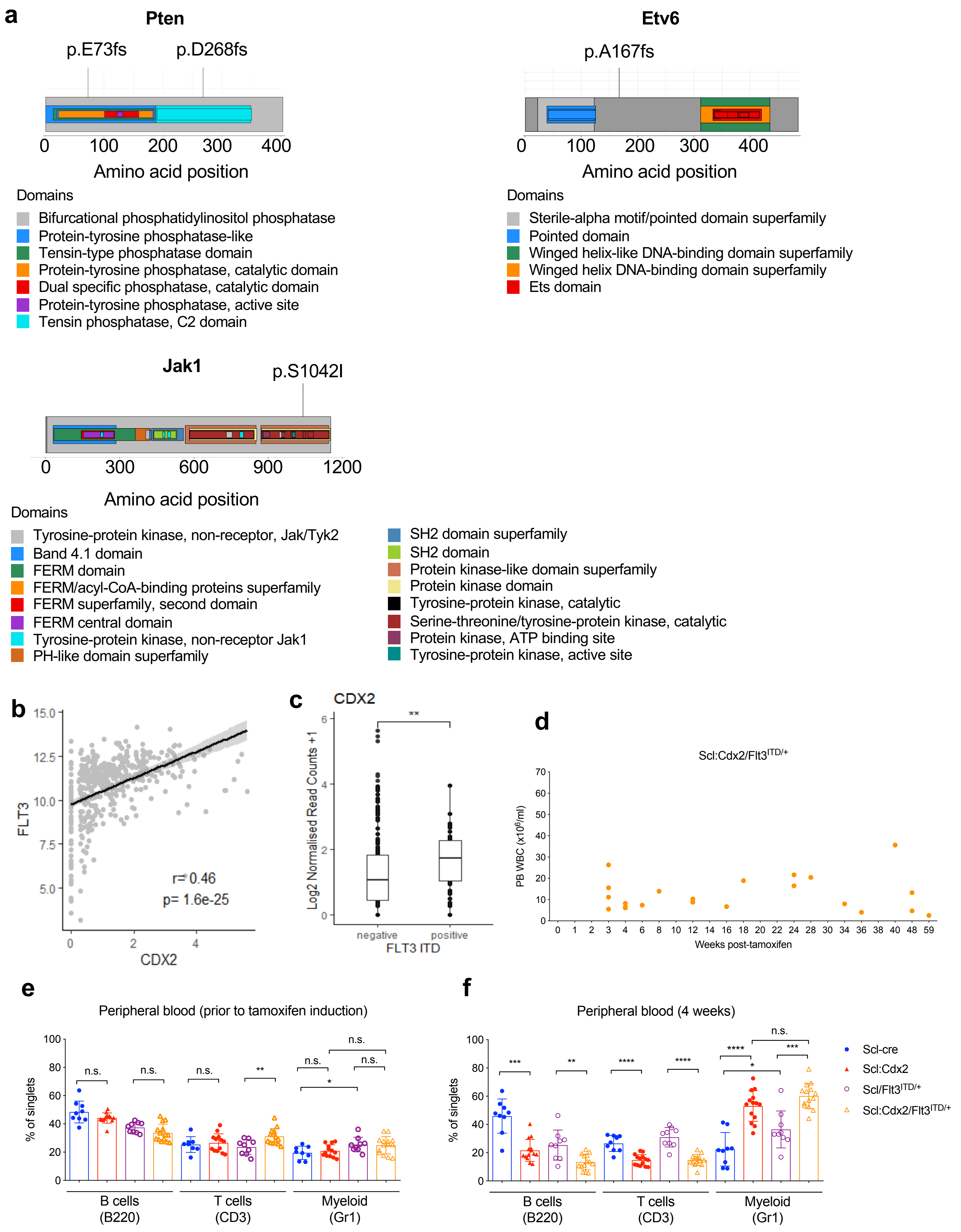

**Supplementary Figure 3. Cdx2 synergizes with Flt3-ITD to accelerate myeloproliferation.**

(a) Lolliplot visualizing somatic mutations in relation to the protein domains of the coding gene. (b) CDX2 and FLT3 co-expression in Beat AML patient cohort[1] (n=461). (c) CDX2 read counts in FLT3-ITD-positive (n=105) compared to FLT3-ITD-negative samples (n=355). Box of the boxplots display the median, $25^{th}$ and $75^{th}$ percentiles. The whiskers extend 1.5x interquartile range from the $25^{th}$ and $75^{th}$ percentile. Data points beyond the range are displayed as individual outlying points. (d) WBC counts of Scl:Cdx2/$Flt3^{ITD/+}$ mice at date of sacrifice (n=22). (e) Frequency of B220-positive B cells, CD3-positive T cells and Gr1-positive myeloid cells in PB of mice prior to tamoxifen induction and (f) four weeks after start of tamoxifen induction (*Scl*-cre n=9; Scl:Cdx2 n=14; Scl/$Flt3^{ITD/+}$ n=9; Scl:Cdx2/$Flt3^{ITD/+}$ n=14). N = biologically independent animals. For (b) r=Pearson's product moment correlation coefficient, p-value is derived from a test for association between paired samples, using one of Pearson's product moment correlation coefficient. Statistical analyses for (c), (e), (f) performed using two-tailed Mann-Whitney test. Data are plotted as mean values +/- SD. n.s.; not significant. * $P < 0.05$, ** $P < 0.01$, *** $P < 0.001$, ****, $P < 0.0001$.

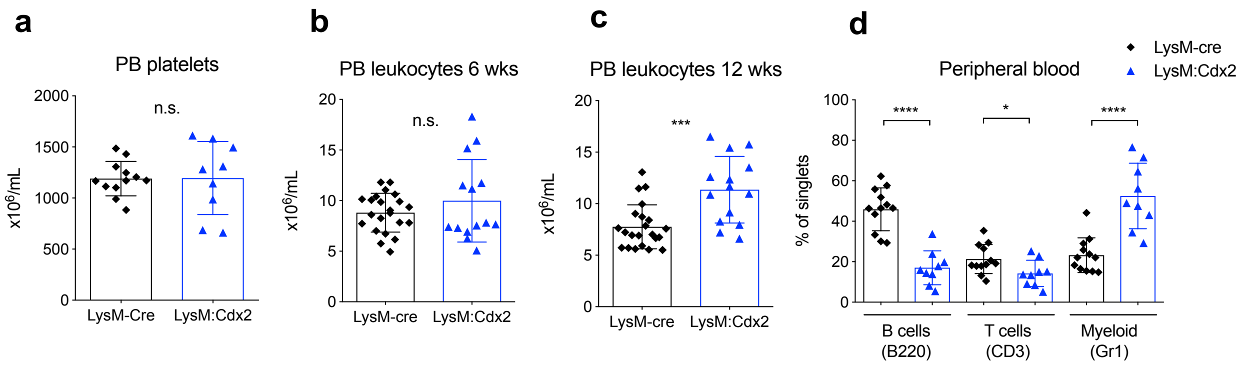


**Supplementary Figure 4. Restricted myeloid expression of Cdx2 causes disease distinct from Scl:Cdx2.**

(a) PB platelet counts of mice at sacrifice (*LysM*-Cre n=12; LysM:Cdx2 n=9). (b) PB leukocyte counts of mice at six weeks and (c) 12 weeks of age (*LysM*-Cre n=22; LysM:Cdx2 n=14). (d) Frequency of B220-positive B cells, CD3-positive T cells and Gr1-positive myeloid cells in PB of mice at sacrifice (*LysM*-Cre n=12; LysM:Cdx2 n=9). N = biologically independent animals. Statistical analyses of graphs performed using two-tailed Mann-Whitney test. Data are plotted as mean values +/- SD. n.s.; not significant. * $P < 0.05$, ** $P < 0.01$, *** $P < 0.001$, ****, $P < 0.0001$.

# Supplementary Figure 5

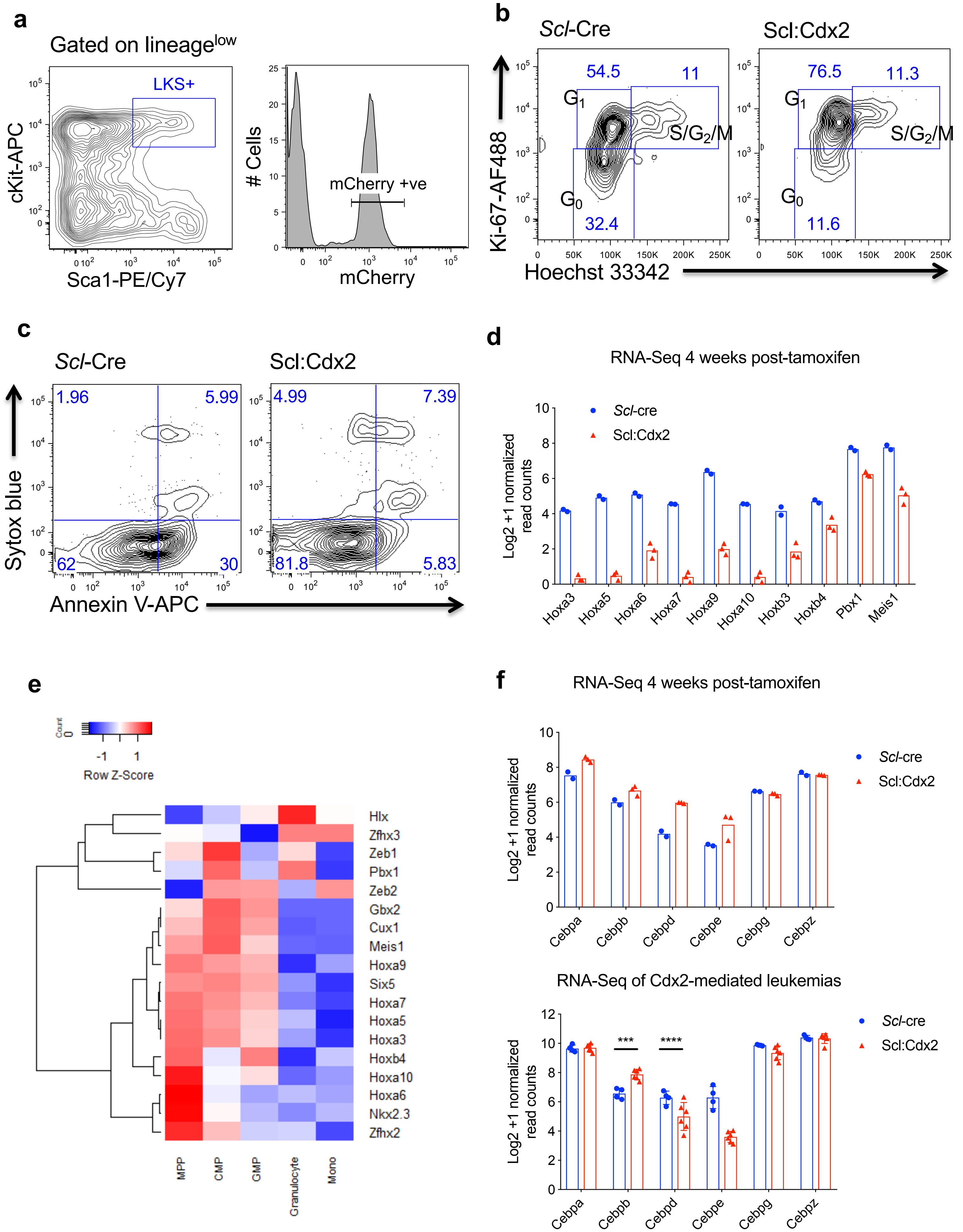

**Supplementary Figure 5. Cdx2 confers a progenitor gene signature associated with differentiation.**

(a) Gating strategy for LKS+ mCherry+ cells used for RNA-Seq. (b) Representative flow cytometry plots of cell cycle and (c) apoptosis assays. (d) Log2 +1 normalized read counts of Homeobox (Hox) family genes comparing *Scl*-Cre and Scl:Cdx2, 4 weeks after tamoxifen (*Scl*-Cre n=2; Scl:Cdx2 n=3). (e) Heatmap of RNA-Seq analysis of sorted multipotent progenitors (MPP), CMP, GMP, granulocytes and monocytes (mono)[1] showing relative expression of Hox genes. Color scale represents Z-score after row normalization. (f) Log2 +1 normalized read counts of Cebp transcripts in pre-leukemic Cdx2 HSCs, 4 weeks after tamoxifen (*Scl*-Cre n=2; Scl:Cdx2 n=3) and in established leukemias (*Scl*-Cre n=4; Scl:Cdx2 n=6). N = biologically independent animals. Statistical analyses performed using False Discovery Rate (FDR) corrected P-values derived from likelihood ratio test of the binomial generalized log-linear modelled gene expression data as implemented in edgeR R package. Data are plotted as mean values +/- SD, *** $P < 0.001$, ****, $P < 0.0001$.

# Supplementary Figure 6

**a**

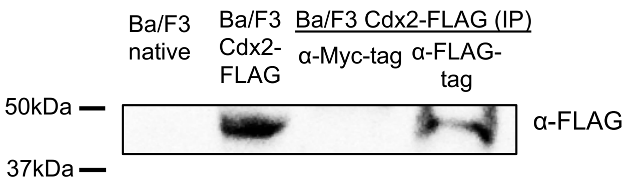


**b**

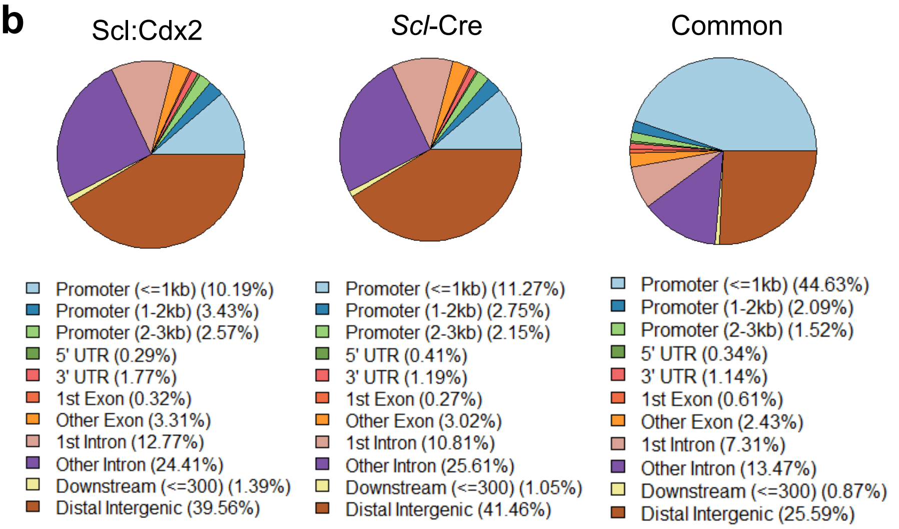


**c**

Known motif enrichment in Cdx2 specific distal elements

| Peaks | Transcription factor | Source | Motif | FDR |
|---|---|---|---|---|
| Scl:Cdx2 | Cdx2 | MC1 ES cells GSE14586 | 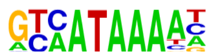 | <.0001 |
| Scl:Cdx2 | Cebpb | Peritoneal macrophages GSM537985 | 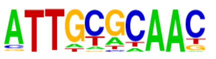 | .01 |

**d**

Distal element peak overlap

| Peaks | CEBPα GMP peak overlap GSM1187163 | No overlap |
|---|---|---|
| *Scl*-Cre | 92 (10.1%) | 813 (89.9%) |
| Scl:Cdx2 | 5738 (39.6%) | 8746 (60.4%) |

Chi-Square test p<0.0001

**e**

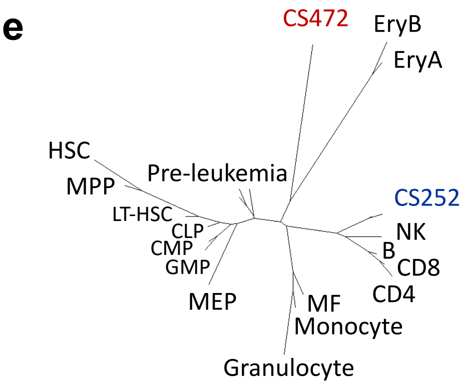


**f**

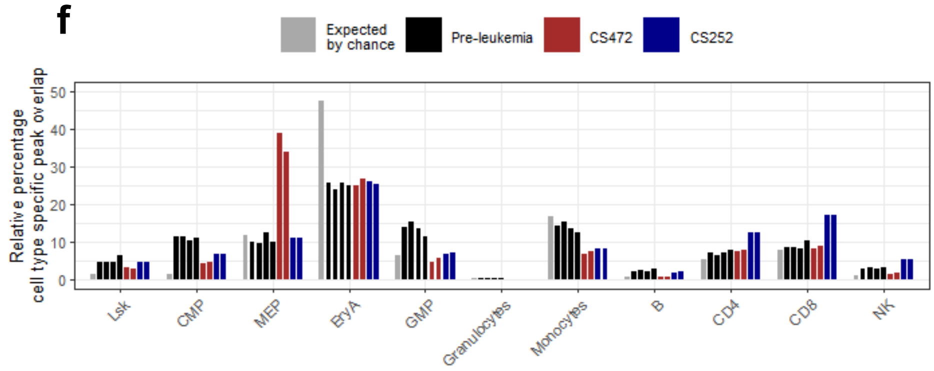


**g**

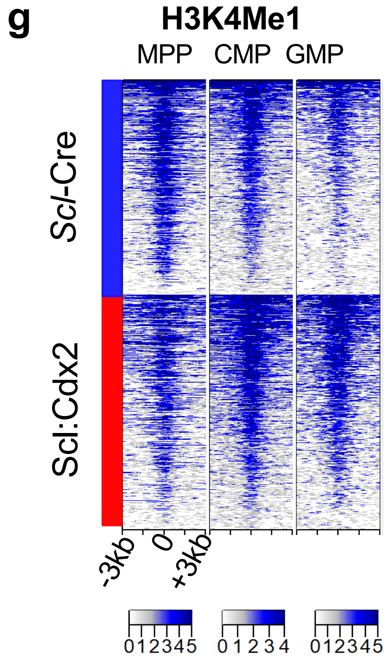


**h**

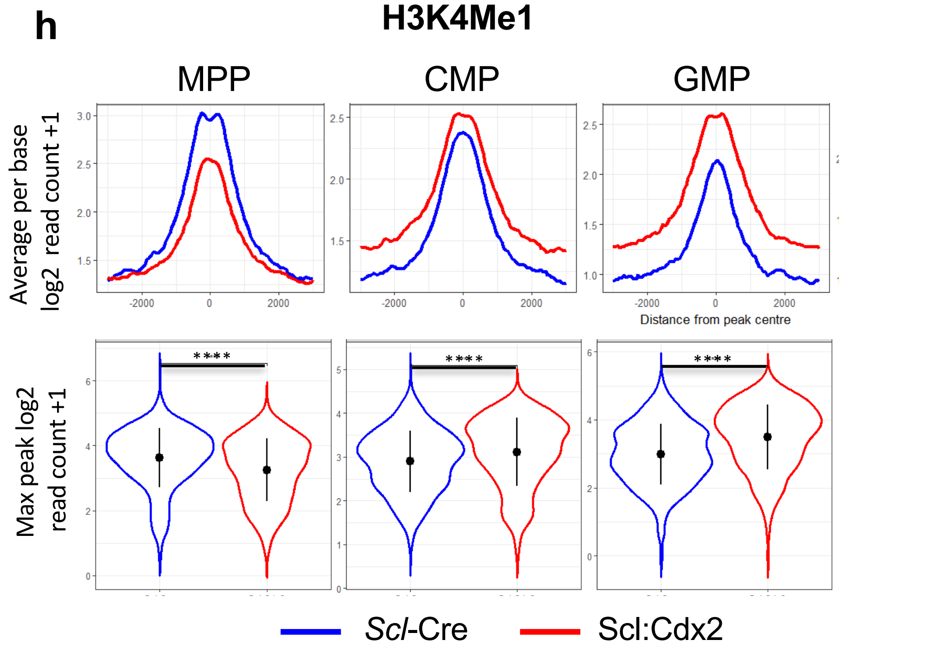

**Supplementary Figure 6. Cdx2 modifies chromatin access in regions with critical differentiation factors.**

(a) Immunoblot of Ba/F3 cells transduced with Cdx2-FLAG and immunoprecipitated with rabbit anti-FLAG tag or rabbit anti-Myc tag (negative control), probed with mouse anti-FLAG antibody. Image contains representative samples from independent cell line replicates, full image supplied in Supplementary Data 6. (b) Proportion of peaks in genomic regions unique to *Scl*-Cre BM LKS+ and unique to Scl:Cdx2 BM LKS+ and common between them. (c) Known motifs identified in Scl:Cdx2 gained distal region peaks by HOMER[3]. (d) Distal element peak overlap between *Scl*-Cre or Scl:Cdx2 ATAC-Seq with publicly available CEBPα GMP ChIP-Seq. (e) RNA-Seq and ATAC-Seq integrated data: Unrooted tree estimate from neighborhood joining of the first 5 principle components of the Cdx2-driven pre-leukemic, T-leukemic (CS252) and erythro-myeloid leukemic (CS472) LKS gene expression, with published hematopoietic cell populations. (f) Relative proportion of overlap in LKS chromatin accessibility with cell type defining peaks, bar graphs. (g) Read coverage heatmaps of MPP, CMP, GMP H3K4Me1 ChIP-Seq[2] overlapping Scl:Cdx2 and *Scl*-Cre specific distal regions. Color scale shows Log2 +1 normalized read counts. (h) Average peak profile (top) and maximum peak height distribution (bottom) summarizing (g). FDR, false discovery rate. Statistical analyses of (h) performed using two-tailed Mann-Whitney test without adjustment for multiple comparisons.  **** $P < 0.0001$.

# Supplementary Figure 7

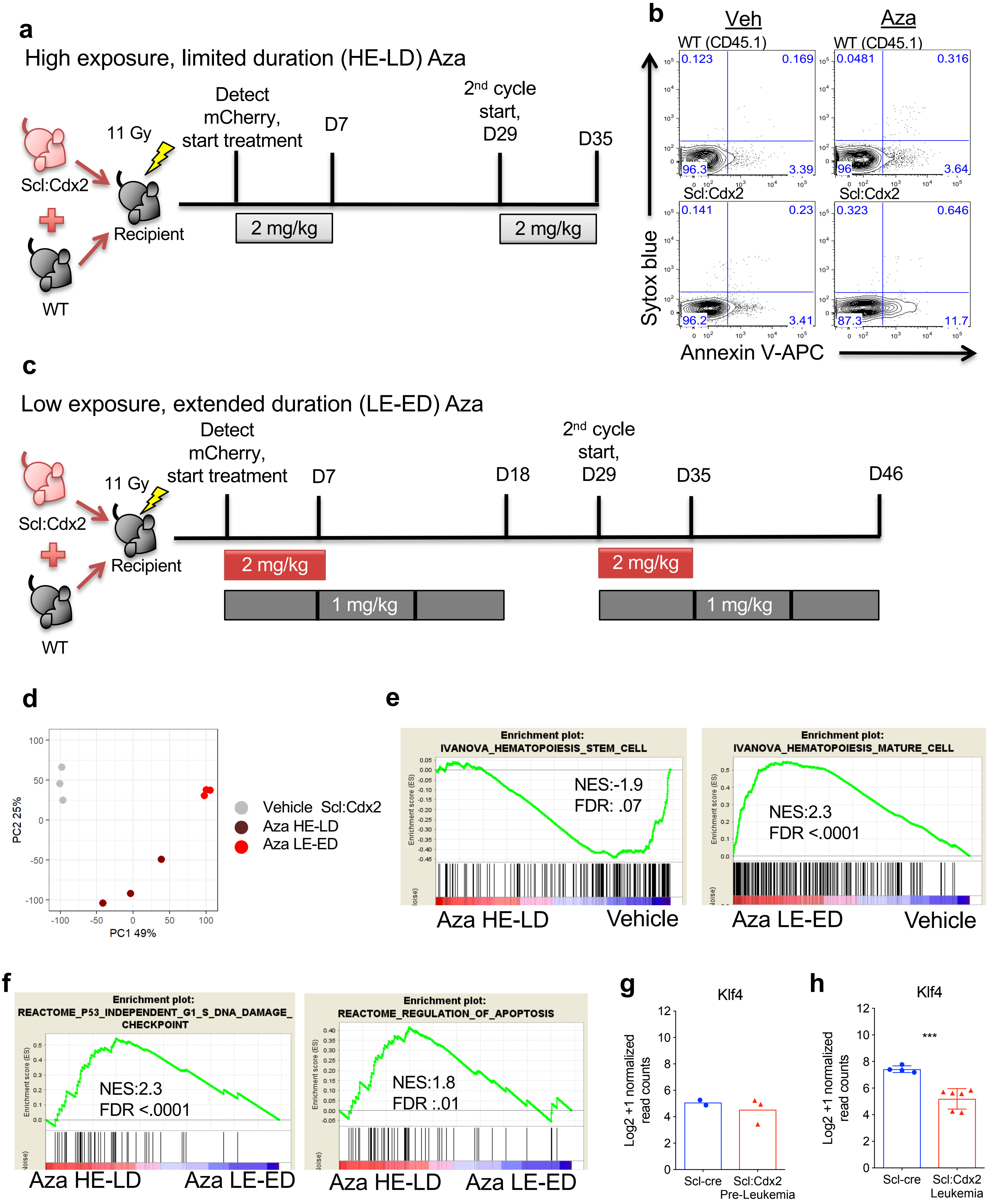

**Supplementary Figure 7. Cdx2-mediated leukemia is responsive to treatment with 5-azacitidine.**

(a) Azacitidine (Aza) treatment experimental schematic. Scl:Cdx2 leukemia cells (CD45.2) and WT (CD45.1) BM cells are mixed and injected into lethally irradiated WT recipients to generate Cdx2 leukemic BM chimeras. When PB mCherry+ cells are detected, mice are treated with 0.9% saline (Veh) or 2 mg/kg Aza for 7 days followed by 21 days rest (high exposure, limited duration; HE-LD). This treatment cycle is repeated a second time until mice reach the euthanasia criteria. (b) Representative flow cytometry plots of apoptosis assay of Veh or Aza-treated PB cells gated on CD45.1 (WT) or CD45.2 (Scl:Cdx2). (c) Alternative Aza treatment experimental schematic. Scl:Cdx2 leukemia cells (CD45.2) and WT (CD45.1) BM cells are transplanted as in panel (a). When PB mCherry+ cells are detected, mice are treated with 2 mg/kg Aza for 7 days followed by 21 days rest, or vehicle or 1 mg/kg Aza 14 times over 21 days followed by 7 days rest (low exposure, extended duration; LE-ED). These treatment cycles are repeated a second time until mice reach the euthanasia criteria. (d) Principal Component Analysis (PCA) of RNA-Seq analysis performed on BM LKS+ mCherry+ cells from mice treated with vehicle, HE-LD Aza or LE-ED Aza. (e) GSEA plots showing immature HSC signature, (f) DNA damage and apoptosis signatures. (g) Normalized read counts of *Klf4* transcripts in primary *Scl*-Cre (n=2) and Scl:Cdx2 (n=3) BM LKS+ cells four weeks after tamoxifen induction (pre-leukemia), and (h) in BM LKS+ of secondary mice transplanted with *Scl*-Cre (n=4) or one of three different Scl:Cdx2 leukemia cells (#252, #472, #882) (n=6). NES, normalized enrichment score. FDR, false discovery rate. Statistical analyses performed using FDR corrected P-values derived from likelihood ratio test as implemented in the edgeR R package. For GSEA, statistical test is two-sided Kolmogorov-Smirnov analysis with adjustment for multiple comparisons. Data are plotted as mean values +/- SD. *** $P < 0.001$.

**Supplementary Tables**

Supplementary Table 1.

Variants found in whole exome sequencing of Scl:Cdx2 leukaemia BM

| Phenotype | ID | Gene | Consequence | Amino acid change |
|---|---|---|---|---|
| AML | 2259 | Etv6 | Frame shift | A167fs |
| AML | 2259 | Pten | Frame shift | D268fs |
| AML | 2259 | Pten | Frame shift | E73fs |
| AML | 2259 | Fat1 | Missense | V2304A |
| AML | 2259 | Raf1 | Missense | S259P |
| B/T AL | 252 | Jak1 | Missense | S1042I |
| B/T AL | 252 | Ikzf1 | Frame shift | N266fs |
| B/T AL | 252 | Nabp2 | Missense | T155M |
| B/T AL | 252 | Zap70 | Nonsense | W163** |
| B/T AL | 252 | Cgref1 | Missense | M180I |
| AEL | 472 | Trp53 | Loss of heterozygosity | E255D |

Supplementary Table 2. Hematopoietic parameters of mice used in RNA-Seq analysis

| Genotype | PB WBC ($x10^6$/ml) | PB Hb (g/dL) | PB Plt ($x10^6$/ml) | Spleen weight (mg) |
|---|---|---|---|---|
| Scl-CreER$^{T2}$ R1 | 6.36 | 14.9 | 898 | 82 |
| Scl-CreER$^{T2}$ R2 | 9.54 | 15 | 860 | 92 |
| Scl/Flt3$^{ITD/+}$ R1 | 6.64 | 13.1 | 546 | 187 |
| Scl/Flt3$^{ITD/+}$ R2 | 8.68 | 13.2 | 844 | 155 |
| Scl:Cdx2 R1 | 17 | 11.6 | 178 | 384 |
| Scl:Cdx2 R2 | 5.06 | 13.3 | 1092 | 260 |
| Scl:Cdx2 R3 | 3.78 | 13 | 214 | 1343 |
| Scl:Cdx2/Flt3$^{ITD/+}$ R1 | 12.06 | 14.8 | 535 | 282 |
| Scl:Cdx2/Flt3$^{ITD/+}$ R2 | 6.12 | 12.7 | 203 | 373 |
| Scl:Cdx2/Flt3$^{ITD/+}$ R3 | 4.38 | 13 | 171 | 266 |
| Scl:Cdx2/Flt3$^{ITD/+}$ R4 | 11.14 | 15 | 169 | 355 |

Supplementary Table 3. Most enriched genesets in Scl:Cdx2 LKS+ based on RNA-Seq

NES, normalized enrichment score; NOM p-val, nominal p-value; FDR q-val, false discovery rate. Statistical tests described in Subramaniam *et. al*.[4]

| Genesets enriched in Scl:Cdx2 LKS+ | NES | NOM p-val | FDR q-val |
|---|---|---|---|
| MITOCHONDRIAL METABOLISM | | | |
| KEGG_OXIDATIVE PHOSPHORYLATION | -2.77 | 0.0 | 0.0 |
| REACTOME_RESPIRATORY_ELECTRON_TRANSPORT | -2.66 | 0.0 | 0.0 |
| MOOTHA_VOXPHOS | -2.56 | 0.0 | 0.0 |
| WONG_MITOCHONDRIA_GENE_MODULE | -2.4 | 0.0 | 0.0 |
| MOOTHA_MITOCHONDRIA | -2.26 | 0.0 | 0.0 |
| CELL CYCLE | | | |
| REACTOME_DNA_REPLICATION | -2.26 | 0.0 | 0.0 |
| WHITFIELD_CELL_CYCLE_LITERATURE | -2.21 | 0.0 | 0.0 |
| REACTOME_REGULATION_OF_MITOTIC_CELL_CYCLE | -2.19 | 0.0 | 0.0 |
| BENPORATH_PROLIFERATION | -2.11 | 0.0 | 0.001 |
| SCIAN_CELL_CYCLE_TARGETS_OF_TP53_AND_TP73_DN | -2.06 | 0.0 | 0.002 |
| REACTOME_M_G1_TRANSITION | -2.06 | 0.0 | 0.002 |
| REACTOME_SYNTHESIS_OF_DNA | -2.02 | 0.0 | 0.003 |
| HSPC DIFFERENTIATION | | | |
| IVANOVA_HEMATOPOIESIS_LATE_PROGENITOR | -2.66 | 0.0 | 0.0 |
| WANG_IMMORTALIZED_BY_HOXA9_AND_MEIS1_UP | -2.54 | 0.0 | 0.0 |
| IVANOVA_HEMATOPOIESIS_INTERMEDIATE_PROGENITOR | -2.51 | 0.0 | 0.0 |
| RAMALHO_STEMNESS_DN | -2.16 | 0.0 | 0.001 |
| WONG_EMBRYONIC_STEM_CELL_CORE | -2.01 | 0.0 | 0.003 |
| TUMOURIGENESIS | | | |
| YU_MYC_TARGETS_UP | -2.1 | 0.0 | 0.001 |
| VILIMAS_NOTCH1_TARGETS_DN | -2.07 | 0.0 | 0.002 |
| RHODES_UNDIFFERENTIATED_CANCER | -2.03 | 0.0 | 0.003 |

Supplementary Table 4. Most enriched genesets in Scl-Cre LKS+ based on RNA-Seq

NES, normalized enrichment score; NOM p-val, nominal p-value; FDR q-val, false discovery rate. Statistical tests described in Subramaniam *et. al*.[4]

| Genesets enriched in Scl-Cre LKS+ | NES | NOM p-val | FDR q-val |
|---|---|---|---|
| WONG_ADULT_TISSUE_STEM_ MODULE | 2.41 | 0.0 | 0.0 |
| BOSCO_INTERFERON_INDUCED_ANTIVIRAL_MODULE | 2.38 | 0.0 | 0.0 |
| MOSERLE_INFA_RESPONSE | 2.11 | 0.0 | 0.0 |
| IVANOVA_HEMATOPOIESIS_STEM_CELL | 2.27 | 0.0 | 0.0 |
| IVANOVA_HEMATOPOIESIS_STEM_CELL_LONG_TERM | 2.09 | 0.0 | 0.0 |

Supplementary Table 5. Antibodies used in this study

Antibodies diluted 1:100 unless otherwise specified.

| Antibody (clone) | Catalogue No. |
|---|---|
| Annexin V; diluted 1:20 | 640920; Biolegend |
| B220-biotin (RA3-6B2) | 103204; Biolegend |
| B220-PerCp/Cy5.5 (RA3-6B2) | 103236; Biolegend |
| c-Kit-APC (2B8) | 105812; Biolegend |
| c-Kit-PE (2B8) | 105808; Biolegend |
| CD150-Brilliant violet 605 (TC15-12F12.2) | 115927; Biolegend |
| CD16/32-PE (93) | 101308; Biolegend |
| CD34-FITC (RAM34) | 11-0341-85; eBioscience |
| CD3e-biotin (145-2C11) | 100304; Biolegend |
| CD3e-PE/Cy7 (145-2C11) | 100320; Biolegend |
| CD45.1-Alexa Fluor 700 (A20) | 561235; Biolegend |
| CD45.1-PE (A20) | 110708; Biolegend |
| CD45.2-FITC (104) | 109806; Biolegend |
| CD45.2-Horizon V500 (104) | 562129; BD Biosciences |
| CD48-Pacfic blue (HM48-1); diluted 1:200 | 103418; Biolegend |
| CD5-biotin (53-7.3) | 100604; Biolegend |
| Gr-1-APC (RB6-8C5) | 108412; Biolegend |
| Gr-1-biotin (RB6-8C5) | 79750; Biolegend |

| | |
|---|---|
| Hoechst 33342 | B2261; Sigma-Aldrich |
| Ki-67-Alexa Fluor 488 (B56) | 561165; BD Biosciences |
| Mac-1 (Cd11b)-APC/Cy7 (M1/70) | 101226; Biolegend |
| Mac-1 (Cd11b)-biotin (M1/70) | 101204; Biolegend |
| Sca-1-PE/Cy7 (D7) | 122514; Biolegend |
| Sytox blue; diluted 1:1000 | S34857; Life Technologies |
| Ter-119-APC (TER-119) | 116212; Biolegend |
| Ter-119-biotin (TER-119) | 116204; Biolegend |
| Cdx2 (CDX-88); diluted 1:1000 | ab157524; Abcam |
| Actin (C4/actin); diluted 1:5000 | 612656; BD Biosciences |
| Myc-Tag (9B11); diluted 1:1000 | 2276S; Cell Signaling |
| DYKDDDDK Tag (Same as Sigma's Anti-FLAG M2) (D6W5B); diluted 1:100 | 14793S; Cell Signaling |
| DYKDDDDK Tag (Same as Sigma's Anti-FLAG M2) (9A3); diluted 1:1000 | 8146S; Cell Signaling |

## Supplementary Methods

### Chromatin immunoprecipitation (ChIP) sequencing

Bone marrow was isolated from WT mice, RBC lysed and lineage depleted as above. Cells were cultured in RPMI/10% FBS with 10ng/mL G-CSF, 50ng/mL mSCF, 10ng/mL mTPO and 30mM HEPES at a concentration of $1x10^6$ cells per mL. Cells were infected with retrovirus at a MOI of 10 in the presence of 8ug/mL polybrene and spun at 1200 xg at 30ºC for 90 minutes and incubated at 37ºC. Cells were harvested and fixed for ChIP at 48 hours post infection. $5x10^6$ cells were washed with PBS and fixed in 5mL 1% Formaldehyde/PBS, rotating for 10 mins at room temp. Fixation was stopped with 0.125M final concentration of Glycine, rotating for 5 minutes at room temperature. Cells were then washed twice with cold PBS and then lysed with 500uL of Lysis Buffer I (10mM Tris ph8, 85mM KCl, 0.5% NP-40, protease inhibitors) for 5 minutes on ice. Nuclei were then pelleted at 750 xg for 5 minutes at 4ºC and lysed with 130uL Lysis Buffer II (1% SDS, 10mM EDTA, 50mM Tris ph8, protease inhibitors) for 15 minutes on ice. Chromatin was then sheared using a Covaris sonicator (duty factor 10%, PIP 140, cycles/burst 200, time 90 seconds). Debris was cleared from the sonicate by spinning at maximum speed for 10 mins and transferring supernatant to a new tube. Remaining sonicate was diluted to 1100uL with dilution buffer (0.5% TritonX-100, 2mM EDTA, 20mM Tris pH8, 150mM NaCl, protease inhibitors). 100uL was retained as a 10% input sample. Diluted sonicate was pre-cleared with 15uL Pierce protein A/G magnetic beads (Thermo Scientific, 88802) and washed in dilution buffer, rotating at 4ºC for 1 hour. Beads were removed and pre-cleared sonicate transferred to a new tube along with 10uL of rabbit anti-FLAG (Cell Signaling, 14793S) and incubated for 16 hours rotating at 4ºC. 25uL of protein A/G magnetic beads, blocked overnight in dilution buffer with 0.1% BSA, were added to each IP and rotated at 4ºC for 90 minutes. Supernatant was removed and beads washed with a series of buffer (Low Salt Wash: 20mM Tris pH8, 2mM EDTA, 125mM NaCl, 0.05% SDS, 1% Triton X100, protease inhibitors; High Salt Wash: 20mM Tris pH8, 500mM NaCl, 0.05% SDS, 1% Triton X100, protease inhibitors; LiCl Wash: 10mM Tris pH8, 2mM EDTA, 250mM LiCl, 1% NP40, % Sodium Deoxycholate, protease inhibitors; TE Wash: 10mM Tris pH8, 1mM EDTA, protease inhibitors) each for 5 minutes rotating at 4ºC. Immune complexes were then eluted from the beads with 150uL Elution buffer (100mM Sodium Bicarbonate, 1% SDS) for 40mins at 65ºC, vortexing every 10 minutes prior to separation from beads. Input samples were topped up with 50uL of elution buffer. Cross-links were reversed with 6uL 5M NaCl and 2uL 20mg/mL Proteinase K, incubated at 65ºC for 2-16 hours. DNA was purified from the eluate using a

QIAquick PCR purification kit (Qiagen, 28104) prior to quantification using the Qubit DNA HS Assay Kit (ThermoFisher).

DNA libraries were prepared using the NEBNext Ultra II DNA Library Prep Kit for Illumina (New England Biolabs). Libraries were quantified using the Qubit DNA HS Assay Kit and analysed for size distribution using the TapeStation (Agilent) prior to pooling and sequencing using 2x 75 bp paired-end on the NextSeq 550 (Illumina) platform. All primary data are available online (GSE146598 [https://www.ncbi.nlm.nih.gov/geo/query/acc.cgi?acc=GSE146598]).

**Whole exome sequencing**

BM cells from *Scl*-CreER$^{T}$-transplanted and Scl:Cdx2 secondary leukemia mice were sorted for CD45.2 to isolate donor cells, and then mCherry-positive and mCherry-negative fractions. Genomic DNA was isolated from these populations using the QIAGEN DNeasy Tissue kit (QIAGEN), libraries were generated with the Agilent SureSelect mouse exome enrichment kit and sequenced on the Illumina HiSeq 4000 (2 × 100 bp). Sequence reads were adapater trimmed using Cutadapt (version 1.11)[5] and aligned using bwa mem[6] to the GRCh37 assembly. Read alignment with a quality below 15 and supplementary alignments were removed prior to variant analysis. Variants were called with VarScan (version 2.4.2)[7] with mCherry-negative as reference and mCherry-positive as alternate (tumor). Variants were filtered for somatic mutations with a p-value <.05. Mutations were annotated if they were located in simple repeats as predicted by RepeatMasker[8], or if they were common mouse SNPs (dbSNP, version 142). In addition, we used two sources to annotate if mutations in the genes were found to be associated with hematological malignancies. The first source is the MSK-HemePACT v3 panel containing 585 genes and the second source is the COSMIC Cancer Gene Census[9] (CGC) filtered for mutations in genes whose cancers primary site was the hematopoietic and lymphoid tissue. Mutations locations in relation to functional protein domains were visualized using GenVisR R package[10]. Raw data are available at the Sequence Read Archive (SRA) with accession number PRJNA552223 [https://www.ncbi.nlm.nih.gov/bioproject/PRJNA552223].

**RNA sequencing**

To analyze pre-leukemic Scl:Cdx2 and Scl:Cdx2/Flt3$^{ITD/+}$ cells, BM LKS+ mCherry-positive samples were sorted from mice four weeks from the start of two weeks of tamoxifen feed induction alongside controls (*Scl*-CreER$^{T}$ and Scl/Flt3$^{ITD/+}$). To analyze leukemic Scl:Cdx2 cells, BM LKS+ mCherry-positive CD45.2-positive cells were extracted from recipient mice

transplanted with 1 × $10^6$ Scl:Cdx2 transformed BM cells or with *Scl*-CreER$^T$ cells. To analyze azacitidine-treated samples, leukemic Scl:Cdx2 mice were inoculated with vehicle (0.9% saline) or azacitidine qd for seven consecutive days (2 mg/kg) or fourteen days spaced over three weeks (1 mg/kg). BM LKS+ mCherry-positive cells were sorted after one treatment cycle.

RNA was extracted with Arcturus Picopure RNA isolation kit (Thermo Fisher). cDNA libraries were prepared using the NEBNext Ultra RNA Library Prep kit with the polyA selection module (New England Biolabs) and purified with AMPure beads (Agilent). Libraries were quantified with the KAPA Library Quantification Kit for Illumina (Kapa Biosystems) and pooled. Sequencing was performed on the Illumina NextSeq 500 Sequencer High Output mode (1 × 75 bp). Mapping, read counting and annotation was performed as previously described[11]. Data normalization and differential gene expression analysis was performed edgeR from the Bioconductor package[12]. Data was analysed using Gene Set Enrichment Analysis (GSEA)[4]. Threshold for differential expression of genes was based a false discovery rate less than 0.05. All primary data are available online (GSE133829 [https://www.ncbi.nlm.nih.gov/geo/query/acc.cgi?acc=GSE133829]).

**Assay for Transposase Accessible Chromatin (ATAC) Sequencing**

ATAC-Seq protocol was performed as outlined in previously[13] on approximately 3,000 – 20,000 sorted BM LKS+ from *Scl*-CreER$^T$ and Scl:Cdx2 mice four weeks after the start of two weeks tamoxifen feed induction. Samples were sequenced on an Illumina NextSeq 500 High Output (2 × 75 bp). Raw reads of one Scl:Cdx2 and one *Scl*-CreER$^T$ samples were adapter trimmed using Cutadapt[14] and mapped to mouse genome mm9 using bwa mem[6]. Reads with mapping quality < 30 and duplicated reads marked with Picard (http://broadinstitute.github.io/picard/) were removed. Uniquely mapped reads were converted to bigwig and normalized reads per million mapped using bedtools genomecov[15] and bedGraphToBigWig (UCSC: https://genome.ucsc.edu/). Peak calling was performed using MACS2[16] with parameters -q 0.01 --nomodel --shift -100 --extsize 200. Peaks overlapping with the mm9 blacklist region generated by UCSC were removed. Peaks were annotated with R package ChIPseeker[17] with transcription start site (TSS) reaching from -3000 to 3000 to the UCSC mm9 gene model and the org.Mm.eg.db Bioconductor annotation package. Peaks were annotated for the CDX2 motif (MA0465.1) using R package Biostrings matchPWM with default parameters. Sequences of top peaks (904 Scl-Cre, 1000 Scl:Cdx2) in distal regulatory regions called solely in the *Scl*-CreER$^T$ or Scl:Cdx2 condition were extracted in addition to the

top 1000 shared distal peaks as a background. Motif enrichment analysis on these sequences was performed using MEME Suite[18] and Homer[3]. *Scl*-CreER$^T$ and Scl:Cdx2 ATAC-Seq tracks, MACS2 peaks and peaks containing the CDX2 motif, and were visualized in UCSC Genome Browser https://genome.ucsc.edu/s/JasminS/VU_2019_CDX2_ATAC.

**RNA-Seq and ATAC-Seq data integration with existing cell type gene expression and accessible chromatin profile data**

Integration of published RNA-Seq data from murine hematopoietic cells (GSE60101 [https://www.ncbi.nlm.nih.gov/geo/query/acc.cgi?acc=GSE60101])[43] with our data were performed using an empirical Bayes framework as implemented in ComBat from sva R package. The unrooted tree estimation was performed using Neighbourhood joining as implemented in the ape R package on the Euclidean distance of the first five principal components explaining 60% of the variability of the batch corrected data. ATAC-Seq of sorted murine hematopoietic cells with accession GSE59992 [https://www.ncbi.nlm.nih.gov/geo/query/acc.cgi?acc=GSE59992][43] were obtained. Peaks were filtered with as score >7 and a width >30. A peak was deemed specific to a cell type if no other cell type had an overlapping peak with a score higher >3. To estimate the relative percentage between the celltype specific data and our data we calculated the number of overlapping peaks for each cell type and divided it by the total number of overlapping peaks. Since cell type specific peaks ranged from 327 (natural killer cells; NK) to 137,035 (mature Erythroid cells; EryA), we also estimated the expected relative percentage of overlapping peaks if peaks were randomly located.

## Supplementary References


1 Tyner, J. W., Tognon, C. E., Bottomly, D., Wilmot, B., Kurtz, S. E. *et al*. Functional Genomic Landscape of Acute Myeloid Leukaemia. *Nature* **562**, 526-531, (2018).

2 Lara-Astiaso, D., Weiner, A., Lorenzo-Vivas, E., Zaretsky, I., Jaitin, D. A. *et al*. Chromatin State Dynamics During Blood Formation. *Science* **345**, 943-949, (2014).

3 Heinz, S., Benner, C., Spann, N., Bertolino, E., Lin, Y. C. *et al*. Simple Combinations of Lineage-Determining Transcription Factors Prime Cis-Regulatory Elements Required for Macrophage and B Cell Identities. *Mol Cell* **38**, 576-589, (2010).

4 Subramanian, A., Tamayo, P., Mootha, V. K., Mukherjee, S., Ebert, B. L. *et al*. Gene Set Enrichment Analysis: A Knowledge-Based Approach for Interpreting Genome-Wide Expression Profiles. *Proc Natl Acad Sci U S A* **102**, 15545-15550, (2005).

5 Martin, M. Cutadapt Removes Adapter Sequences from High-Throughput Sequencing Reads. *EMBnet.journal; Vol 17, No 1: Next Generation Sequencing Data Analysis*, (2011).

6 Li, H. & Durbin, R. Fast and Accurate Short Read Alignment with Burrows-Wheeler Transform. *Bioinformatics* **25**, 1754-1760, (2009).

7 Koboldt, D. C., Zhang, Q., Larson, D. E., Shen, D., McLellan, M. D. *et al*. Varscan 2: Somatic Mutation and Copy Number Alteration Discovery in Cancer by Exome Sequencing. *Genome research* **22**, 568-576, (2012).

8 Smit, A., Hubley, R. & Green, P. *RepeatMasker Open-4.0*. http://www.repeatmasker.org, (2013-2015).

9 Sondka, Z., Bamford, S., Cole, C. G., Ward, S. A., Dunham, I. *et al*. The Cosmic Cancer Gene Census: Describing Genetic Dysfunction across All Human Cancers. *Nature reviews. Cancer* **18**, 696-705, (2018).

10 Skidmore, Z. L., Wagner, A. H., Lesurf, R., Campbell, K. M., Kunisaki, J. *et al*. Genvisr: Genomic Visualizations in R. *Bioinformatics* **32**, 3012-3014, (2016).

11 Jacquelin, S., Straube, J., Cooper, L., Vu, T., Song, A. *et al*. Jak2v617f and Dnmt3a Loss Cooperate to Induce Myelofibrosis through Activated Enhancer-Driven Inflammation. *Blood*, blood-2018-2004-846220, (2018).

12 Robinson, M. D., McCarthy, D. J. & Smyth, G. K. Edger: A Bioconductor Package for Differential Expression Analysis of Digital Gene Expression Data. *Bioinformatics* **26**, 139-140, (2010).

13 Buenrostro, J. D., Wu, B., Chang, H. Y. & Greenleaf, W. J. Atac-Seq: A Method for Assaying Chromatin Accessibility Genome-Wide. *Curr Protoc Mol Biol* **109**, 21.29.21-21.29.29, (2015).

14 Martin, M. *Cutadapt Removes Adapter Sequences from High-Throughput Sequencing Reads*. Vol. 17 (2011).

15 Quinlan, A. R. & Hall, I. M. Bedtools: A Flexible Suite of Utilities for Comparing Genomic Features. *Bioinformatics* **26**, 841-842, (2010).

16 Zhang, Y., Liu, T., Meyer, C. A., Eeckhoute, J., Johnson, D. S. *et al*. Model-Based Analysis of Chip-Seq (Macs). *Genome biology* **9**, R137-R137, (2008).

17 Yu, G., Wang, L.-G. & He, Q.-Y. Chipseeker: An R/Bioconductor Package for Chip Peak Annotation, Comparison and Visualization. *Bioinformatics* **31**, 2382-2383, (2015).

18 Bailey, T. L., Boden, M., Buske, F. A., Frith, M., Grant, C. E. *et al*. Meme Suite: Tools for Motif Discovery and Searching. *Nucleic Acids Res* **37**, W202-W208, (2009).
