# Supplementary material for: Hematopoietic stem and progenitor cell-restricted Cdx2 expression induces transformation to myelodysplasia and acute leukemia
Source: Nat Commun. 2020 Jun 15;11:3021. doi: 10.1038/s41467-020-16840-2 (PMC7296000; doi:10.1038/s41467-020-16840-2)
Supplement: Supplementary file 3 — Description of Additional Supplementary Files [file 41467_2020_16840_MOESM3_ESM.docx]

**Description of Additional Supplementary Files**

**Supplementary Data 1**

Somatic, nonsynonymous and frameshift mutations, and loss of heterozygosity (LOH) from exome sequencing of Cdx2-expressing (mCherry-positive) leukemic bone marrow versus matched normal (mCherry-negative) bone marrow harvested from the same recipient host. Four independent leukemia samples were sequenced.

**Supplementary Data 2**

Differentially expressed gene list and analysis of *Scl*-cre (n=2) and Scl:Cdx2 (n=3) bone marrow Lineage^low^cKit^+^Sca1^+^ (LKS) cells sorted for mCherry at four weeks after the start of tamoxifen induction. Results are sorted by FDR adjusted P-value. logFC, Log2 fold change; logCPM, average Log2 counts per million; LR, likelihood ratio statistics; PValue, two-sided P-value; adjP (FDR), false discovery rate adjusted P-value.

**Supplementary Data 3**

Differentially expressed gene list either upregulated (UP) or downregulated (DN) in Cdx2-expressing BM LKS mCherry-positive cells versus Ctrl (*Scl*-cre BM LKS). Pre-leukemiae samples represent Scl:Cdx2 cells sequenced four weeks after tamoxifen induction. CS252 represents Scl:Cdx2 #252 (B/T-ALL). CS882 represents Scl:Cdx2 #882 (T-ALL). CS2259 represents Scl:Cdx2 #2259 (AML). CS472 represents Scl:Cdx2 #472 (AEL). Genes are also filtered as being UP in both CS252 and CS882 but not CS2259 and CS472, or DN in both CS252 and CS882 but not CS2259 and CS472, characterizing a transcriptional signature associated with lymphoid malignancy.

**Supplementary Data 4**

Enrichr RNA-Seq analysis of genes significantly upregulated in Scl:Cdx2 lymphoid leukemia samples (from Supplementary Table 4) that show significant overlap with signatures deregulated upon transcription factor alteration in T lymphocytes and T-cell leukemia (red text indicates gene set enrichment with adjusted P-value < 0.05). Additional gene set libraries with gene sets overlapping the shared Scl:Cdx2 lymphoid UP gene list are included if the P-value < 0.05 but the adjusted P-value is not significant.

**Supplementary Data 5**

Differential gene expression analysis of LKS+ mCherry-sorted bone marrow cells, comparing high exposure and limited duration azacitidine treatment (Aza 7 days), low exposure and extended duration azacitidine treatment (Aza 14 days), and Vehicle treatment of Scl:Cdx2 leukemic mice. N = 3 biologically independent animals per group.

**Supplementary Data 6**

Uncropped original scans of immunoblots displayed in Supplementary Figure 1c, anti-CDX2 and anti-𝛃-actin (page 1) and Supplementary Figure 6a, anti-FLAG (page 2). Red boxes indicate part of image used in Supplementary Figures.
