# Supplementary material for: Hematopoietic stem and progenitor cell-restricted Cdx2 expression induces transformation to myelodysplasia and acute leukemia
Source: Nat Commun. 2020 Jun 15;11:3021. doi: 10.1038/s41467-020-16840-2 (PMC7296000; doi:10.1038/s41467-020-16840-2)

Supplementary Data 1 - Uncropped original scans

Supplementary Figure 1c

mouse anti-Cdx2 (CDX-88, 1:1000)

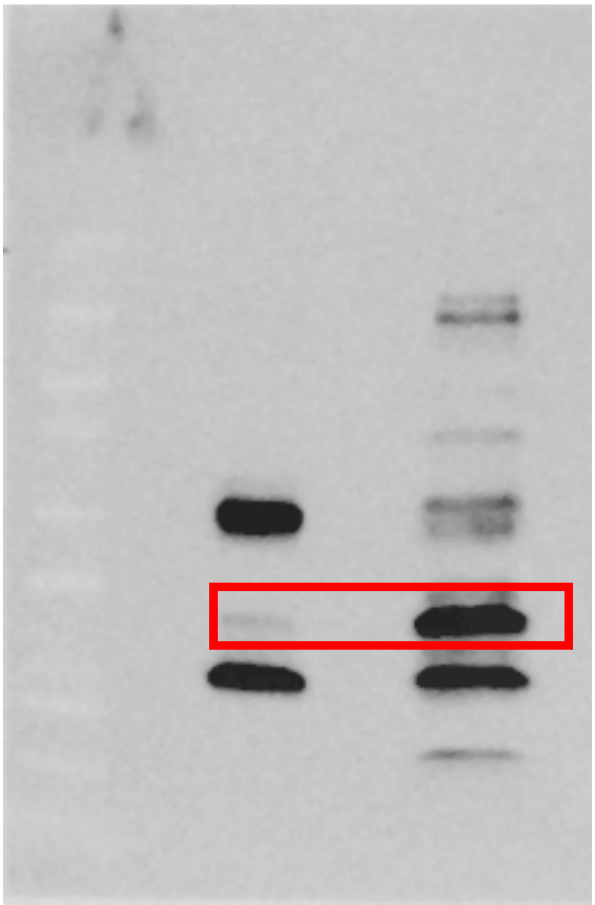

mouse anti-β-Actin (1:5000)

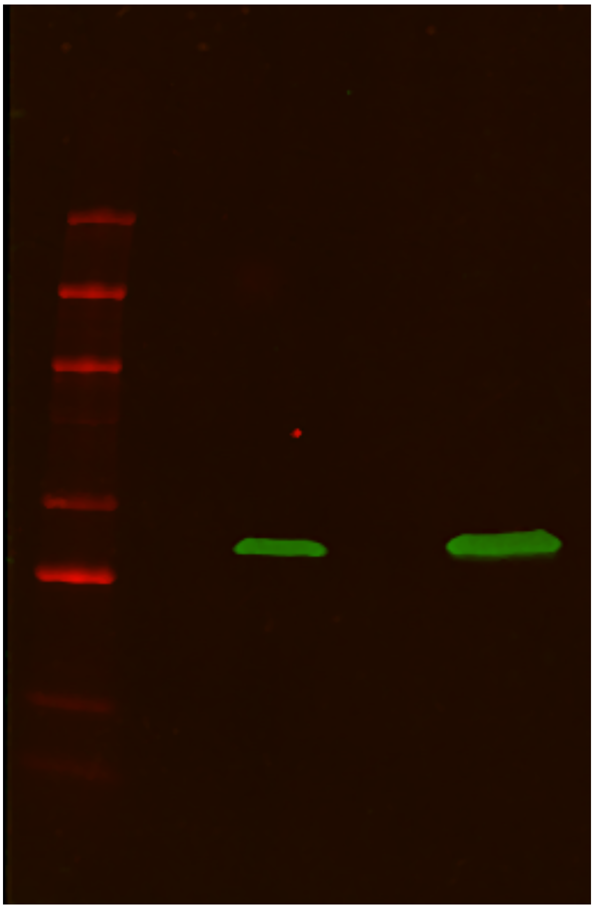

mouse anti-β-Actin (1:5000)

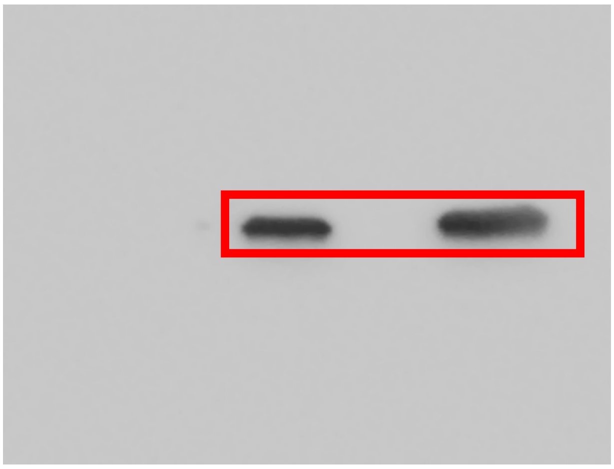

Supplementary Figure 6a

mouse anti-Flag (1:1000)

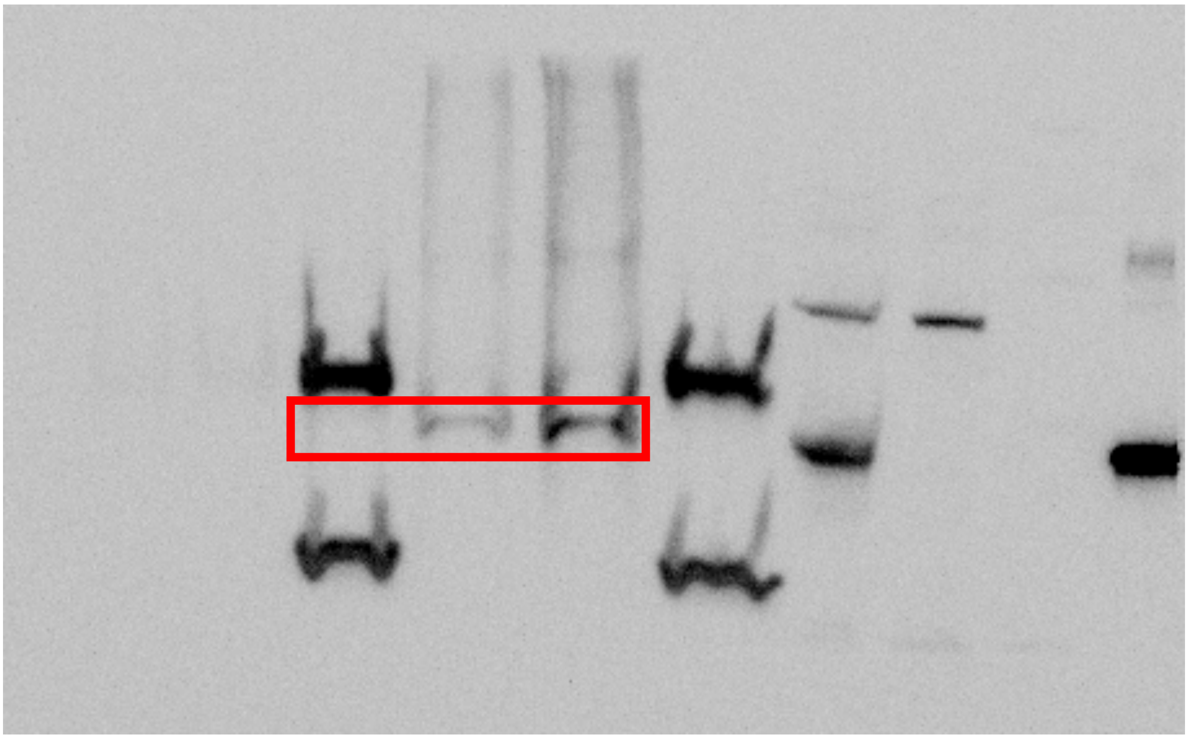

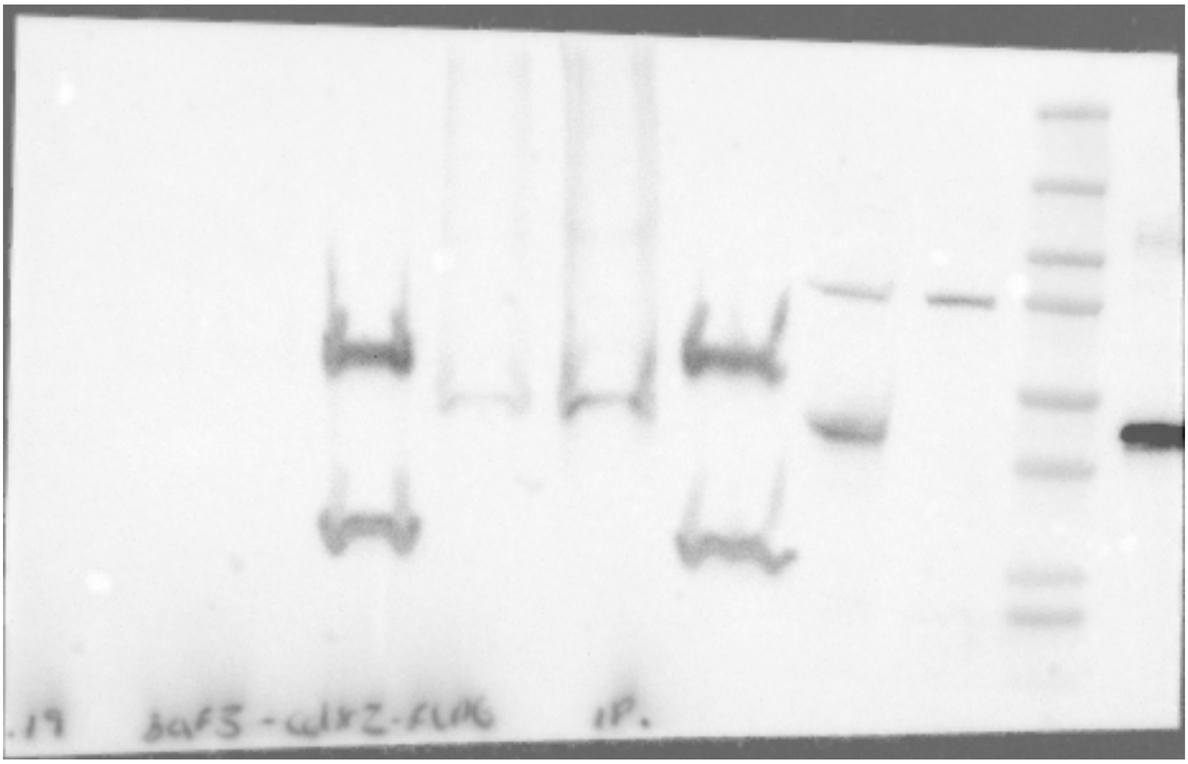

Supplement: Supplementary file 9 — Supplementary Data 6 [file 41467_2020_16840_MOESM9_ESM.pdf]
