# Supplementary material for: Hematopoietic stem and progenitor cell-restricted Cdx2 expression induces transformation to myelodysplasia and acute leukemia
Source: Nat Commun. 2020 Jun 15;11:3021. doi: 10.1038/s41467-020-16840-2 (PMC7296000; doi:10.1038/s41467-020-16840-2)
Supplement: Supplementary file 10 — Reporting Summary [file 41467_2020_16840_MOESM10_ESM.pdf]

nature research

Corresponding author(s): Steven Lane

Last updated by author(s): May 17, 2020

Reporting Summary

Nature Research wishes to improve the reproducibility of the work that we publish. This form provides structure for consistency and transparency in reporting. For further information on Nature Research policies, see Authors & Referees and the Editorial Policy Checklist .

Statistics

For all statistical analyses, confirm that the following items are present in the figure legend, table legend, main text, or Methods section.

n/a	Confirmed	
☐	☒	The exact sample size (*n*) for each experimental group/condition, given as a discrete number and unit of measurement
☐	☒	A statement on whether measurements were taken from distinct samples or whether the same sample was measured repeatedly
☐	☒	The statistical test(s) used AND whether they are one- or two-sided<br>*Only common tests should be described solely by name; describe more complex techniques in the Methods section.*
☒	☐	A description of all covariates tested
☐	☒	A description of any assumptions or corrections, such as tests of normality and adjustment for multiple comparisons
☐	☒	A full description of the statistical parameters including central tendency (e.g. means) or other basic estimates (e.g. regression coefficient) AND variation (e.g. standard deviation) or associated estimates of uncertainty (e.g. confidence intervals)
☐	☒	For null hypothesis testing, the test statistic (e.g. *F*, *t*, *r*) with confidence intervals, effect sizes, degrees of freedom and *P* value noted<br>*Give P values as exact values whenever suitable.*
☒	☐	For Bayesian analysis, information on the choice of priors and Markov chain Monte Carlo settings
☒	☐	For hierarchical and complex designs, identification of the appropriate level for tests and full reporting of outcomes
☒	☐	Estimates of effect sizes (e.g. Cohen's *d*, Pearson's *r*), indicating how they were calculated

Our web collection on statistics for biologists contains articles on many of the points above.

Software and code

Policy information about availability of computer code

Data collection	All flow cytometry data was acquired with FACSDiva software v8.0.1. qRT-PCR reactions were performed on a Applied Biosystems QuantStudio 5 Real-Time PCR System. RNA-Seq and ATAC-Seq libraries were sequenced on the Illumina NextSeq 500 Sequencer High Output mode (1x 75bp, 2x 75bp respectively). ChIP-Seq libraries were sequenced on the Illumina NextSeq550 Sequencer (2x 75bp).
Data analysis	All flow cytometry analysis was performed using FlowJo v9.9.6 (Tree Star). All graphs were drawn in GraphPad Prism v6.0 and v7.0. RNA-Seq sample processing and analysis used the following software: Cutadapt v1.11, edgeR R package v3.26.8, GSEA v2.2 (Broad Institute). Integration of published RNA-Seq data with our data was performed using an empirical Bayes framework as implemented in ComBat from sva R package v3.32.1 and Neighbourhood joining in the ape R package v5.3. ATAC-Seq and ChIPSeq data analysed using the following software: Cutadapt v 1.11, BWA v0.7.15, Picard v2.19, bedtools v2.29, MACS2 v2.10.20150731, ChIPSeeker R package v1.20, org.Mm.eg.db Bioconductor annotation package v3.8.2, matchPWM function of Biostrings R package v2.52, MEME Suite v5.1.1 and HOMER v4.8. ExomeSeq: Cutadapt v 1.11, BWA mem v0.7.15, VarScan v2.4.2, RepeatMasker (open-v4.0), dbSNP v142, GenVisR v1.16.1

For manuscripts utilizing custom algorithms or software that are central to the research but not yet described in published literature, software must be made available to editors/reviewers. We strongly encourage code deposition in a community repository (e.g. GitHub). See the Nature Research guidelines for submitting code & software for further information.

Data

Policy information about availability of data

All manuscripts must include a data availability statement. This statement should provide the following information, where applicable:

- Accession codes, unique identifiers, or web links for publicly available datasets
- A list of figures that have associated raw data
- A description of any restrictions on data availability

The RNA-Seq datasets generated and analysed during the current study are available in the GEO database (https://www.ncbi.nlm.nih.gov/geo) under the SuperSeries accession number GSE133829 (GSE133679, BM LKS four weeks after tamoxifen treatment; GSE133680, Cdx2-mediated AML treated with Azacitidine or vehicle; GSE133828, Cdx2-mediated acute leukemia BM LKS). Publicly available datasets published by Lara-Astiaso et al.43 was obtained from the GEO database,

accession numbers GSE60101 (RNA-Seq) and GSE59992 (ATAC-Seq). Publicly available dataset for Cebpa ChIP-Seq on mouse GMP was obtained from GEO database, accession number GSM1187163.The ChIP-Seq dataset generated during this study on Cdx2-FLAG-transduced mouse BM is available at the accession number GSE146598. The ATAC-Seq dataset performed on BM LKS four weeks after tamoxifen treatment can be accessed here: https://genome.ucsc.edu/s/JasminS/VU_2019_CDX2_ATAC. Whole exome sequencing (WES) datasets performed on Cdx2 mouse BM are available at the Sequence Read Archive (SRA) with accession number PRJNA552223. Whole exome sequencing data for Supplementary Figure 3A and Table 1 can be found in Supplementary Table 1. RNA-Seq data for Figures 5A-H and Supplementary Figures 5D-F can be found in Supplementary Table 2. RNA-Seq data for Figures 7J-M, Supplementary Figure 5F and Supplementary Figure 7D-G can be found in Supplementary Table 5. All other data supporting the findings of this study are available within the article and its supplementary information files and from the corresponding authors upon reasonable request.

Field-specific reporting

Please select the one below that is the best fit for your research. If you are not sure, read the appropriate sections before making your selection.

[x] Life sciences   [ ] Behavioural & social sciences   [ ] Ecological, evolutionary & environmental sciences

For a reference copy of the document with all sections, see nature.com/documents/nr-reporting-summary-flat.pdf

Life sciences study design

All studies must disclose on these points even when the disclosure is negative.

Sample size	No sample-size calculation was performed however mouse experiments were conducted with a minimum number of animals while satisfying good statistical design, in accordance with the Australian code for animal ethics. A minimum of three biological replicates were used per experimental condition except for RNA-Sequencing where two groups had two replicates and ChIP-Sequencing where one sample was used and internally controlled.
Data exclusions	No data was excluded from analyses.
Replication	All in vivo animal experiments were repeated at least twice to confirm reproducibility. All attempts at replication were successful.
Randomization	Mice were bred in-house and experimental groups were populated with mice as soon as they became available. Mice were randomly assigned to groups where applicable. Experimental groups were age and sex matched in each experiment.
Blinding	Blinding is not relevant to the current study as animals were assigned to experiments based on genotype with the exception of azacitidine experiments where mice were randomly assigned prior to treatment. Investigators were blinded to sample identity when scoring disease in mice and quantifying leukaemic blasts and hypersegmented neutrophil nuclei in mouse peripheral blood smears.

Reporting for specific materials, systems and methods

We require information from authors about some types of materials, experimental systems and methods used in many studies. Here, indicate whether each material, system or method listed is relevant to your study. If you are not sure if a list item applies to your research, read the appropriate section before selecting a response.

Materials & experimental systems

n/a	Involved in the study	
[ ]	[x]	Antibodies
[x]	[ ]	Eukaryotic cell lines
[x]	[ ]	Palaeontology
[ ]	[x]	Animals and other organisms
[x]	[ ]	Human research participants
[x]	[ ]	Clinical data

Methods

n/a	Involved in the study	
[ ]	[x]	ChIP-seq
[ ]	[x]	Flow cytometry
[x]	[ ]	MRI-based neuroimaging

Antibodies

Antibodies used

Annexin V 640920; Biolegend
β-actin 612656; BC Biosciences
B220-biotin (RA3-6B2) 103204; Biolegend
B220-PerCp/Cy5.5 (RA3-6B2) 103236; Biolegend
c-Kit-APC (2B8) 105812; Biolegend
c-Kit-PE (2B8) 105808; Biolegend
Cd150-Brilliant violet 605 (TC15-12F12.2) 115927; Biolegend
Cd16/32-PE (93) 101308; Biolegend
Cd34-FITC (RAM34) 11-0341-85; eBioscience
Cd3e-biotin (145-2C11) 100304; Biolegend

Cd3e-PE/Cy7 (145-2C11) 100320; Biolegend
CD45.1-Alexa Fluor 700 (A20) 561235; Biolegend
CD45.1-PE (A20) 110708; Biolegend
CD45.2-FITC (104) 109806; Biolegend
CD45.2-Horizon V500 (104) 562129; BD Biosciences
Cd48-Pacfic blue (HM48-1) 103418; Biolegend
Cd5-biotin (53-7.3) 100604; Biolegend
Gr-1-APC (RB6-8C5) 108412; Biolegend
Gr-1-biotin (RB6-8C5) 79750; Biolegend
Hoechst 33342 B2261; Sigma-Aldrich
Ki-67 (B56) 561165; BD Biosciences
Mac-1 (Cd11b)-APC/Cy7 (M1/70) 101226; Biolegend
Mac-1 (Cd11b)-biotin (M1/70) 101204; Biolegend
Sca-1-PE/Cy7 (D7) 122514; Biolegend
Sytox blue S34857; Life Technologies
Ter-119-APC (TER-119) 116212; Biolegend
Ter-119-biotin (TER-119) 116204; Biolegend
Cdx2 (CDX-88) ab157524; Abcam
Actin (C4/actin) 612656; BD Biosciences
Myc-Tag (9B11) 2276S; Cell Signaling
DYKDDDDK Tag (Same as Sigma's Anti-FLAG M2) (D6W5B) 14793S; Cell Signaling
DYKDDDDK Tag (Same as Sigma's Anti-FLAG M2) (9A3) 8146S; Cell Signaling

Validation

All antibodies were validated by the supplier for mouse samples and for the application used in the study.

Animals and other organisms

Policy information about studies involving animals; ARRIVE guidelines recommended for reporting animal research

Laboratory animals

For transplant recipients 6-8 week old C57BL/6J or C57BL6/J x Ptprca male and female were bred and maintained at QIMR Berghofer Medical Research Institute. Mice were age and sex-matched between groups in all experiments. LSL-Cdx2-mCherry transgenic mice were generated by TaconicArtemis. Flt3-ITD mice were obtained from Dr Wallace Langdon. Scl-CreERT mice were obtained from Dr Carl Walkey. LysM-Cre mice were obtained from Jackson Laboratories. In non-transplant experiments transgenic mice were monitored and phenotyped for clinical pathology up to 2 years of age. Both males and females were used. All mice were bred and maintained at QIMR Berghofer Medical Research Institute.

Wild animals

This study did not involve wild animals.

Field-collected samples

This study did not involve field-collected samples.

Ethics oversight

All procedures were performed in accordance with the QIMR Berghofer Animal Ethics Committee (A11605M).

Note that full information on the approval of the study protocol must also be provided in the manuscript.

ChIP-seq

Data deposition

- [x] Confirm that both raw and final processed data have been deposited in a public database such as GEO.
- [x] Confirm that you have deposited or provided access to graph files (e.g. BED files) for the called peaks.

Data access links
May remain private before publication.

Raw data generated from ChIP-Seq in this study is deposited in GEO database under accession code GSE146598

Files in database submission

Cdx2_Flag_1.fq.gz
Cdx2_Flag_2.fq.gz
Cdx2_Input_1.fq.gz
Cdx2_Input_2.fq.gz
EV_Flag_1.fq.gz
EV_Flag_2.fq.gz
EV_Input_1.fq.gz
EV_Input_2.fq.gz

Genome browser session
(e.g. UCSC)

https://genome.ucsc.edu/s/JasminS/VU_2019_CDX2_ATAC

Methodology

Replicates	For ChIP-Seq, one ChIP sample per group was paired with one Input sample, for validating and supporting the findings of ATAC-Seq data.
Sequencing depth	Over 200 Million unique and paired reads were mapped per sample with q>30
Antibodies	Myc-Tag (9B11) 2276S; Cell Signaling<br>DYKDDDDK Tag (Same as Sigma's Anti-FLAG M2) (D6W5B) 14793S; Cell Signaling<br>DYKDDDDK Tag (Same as Sigma's Anti-FLAG M2) (9A3) 8146S; Cell Signaling
Peak calling parameters	Peaks were called with MACS and parameters -q 0.01 --nomodel --shift -100 --extsize 200
Data quality	Of sequenced reads >92% had >Q30 with a mean quality score of 34
Software	ChIPSeq data was analysed using the following software: Cutadapt, BWA, Picard, bedtools, MACS2, ChIPSeeker R package, Biostrings, matchPWM R package, MEME Suite and HOMER

Flow Cytometry

Plots

Confirm that:

- [x] The axis labels state the marker and fluorochrome used (e.g. CD4-FITC).
- [x] The axis scales are clearly visible. Include numbers along axes only for bottom left plot of group (a 'group' is an analysis of identical markers).
- [x] All plots are contour plots with outliers or pseudocolor plots.
- [x] A numerical value for number of cells or percentage (with statistics) is provided.

Methodology

Sample preparation	Mouse bone marrow was harvested from femurs, tibias and pelvis bones, flushed through a 261/2 gauge needle and filtered through a 70 micron filter with PBS, 2% FBS. Mouse spleen cells were dissected and filtered through a 70 micron filter using a syringe plunger and PBS, 2% FBS. Peripheral blood was collected from the retro-orbital vein by capillary tube into EDTA-coated vials. All cells were washed in PBS, 2% FBS and red blood cell lysed prior to flow cytometry.
Instrument	BD LSRII Fortessa and BD FACSAriaIII
Software	BD FACS Diva (BD Biosciences) and FlowJo (Tree Star)
Cell population abundance	A minimum of 20, 000 events were collected within post-sort fractions for RNA-Seq, ATAC-Seq and whole exome sequencing samples. Post-sort analysis determined that sample purity was at least over 95%.
Gating strategy	Cells were first gated based on morphology on a FSC-A/SSC-A dot plot to exclude dead cells and debris. FSC threshold was set at 30,000 to avoid collecting these events. Positive mCherry events in Cdx2 samples were gated based on known mCherry-negative samples.

- [x] Tick this box to confirm that a figure exemplifying the gating strategy is provided in the Supplementary Information.
